# Metagenomic surveillance of tick-borne pathogens and microbiomes in Huntingdon County, Pennsylvania

**DOI:** 10.1016/j.onehlt.2025.101305

**Published:** 2025-12-18

**Authors:** Andrew Buonaccorsi, Brittney N. McMullen, Brie Builder, Kelliann Drummond, Sarah Halteman, Jeremy Chen See, Evan Thomas, Alexa Viands, Sarah Worley, Justin R. Wright, Jill Keeney, Regina Lamendella

**Affiliations:** a Department of Biology, Juniata College, Huntingdon, PA, USA; b Contamination Source Identification, LLC, Huntingdon, PA, USA; c Department of Communication and Center for Community Engagement, Juniata College, Huntingdon, PA, USA

**Keywords:** Tick, Tick-borne pathogens, Microbiome, PCR, Metagenomics

## Abstract

The rise in tick populations across the United States has contributed to a surge in tick-borne diseases, with Pennsylvania ranking among the highest in reported cases. To better understand local pathogen prevalence and microbial community structure, an integrative study of ticks collected from ten recreational trails in Huntingdon County, Pennsylvania during the summer of 2023 was conducted. A total of 96 ticks were sampled, with 33 PCR-positive specimens selected for shotgun metagenomic sequencing. Pathogen screening via qPCR detected *Borreliella burgdorferi*, *Borrelia miyamotoi*, *Babesia* spp., and *Anaplasma phagocytophilum*. Shotgun metagenomics revealed a broader diversity of tick-borne pathogens, including *Rickettsia* and *Ehrlichia* spp., and demonstrated increased sensitivity by detecting low-abundance pathogens in samples that were PCR-negative. Co-infections were common, and multivariate statistical analysis identified significant associations between environmental variables (e.g., humidity, time of day, land cover) and microbial diversity and predicted gene function. Notably, diversity was higher in ticks collected during early afternoon and from northern sites. Co-occurrence network analysis showed *Rickettsia* as a central taxon with multiple significant positive associations with other microbes while other pathogens were largely absent or peripheral. These findings underscore the enhanced resolution of metagenomic approaches for pathogen detection and the value of combining molecular surveillance with ecological metadata. Our study provides critical insights into local tick microbiomes and pathogen prevalence, which may inform public health interventions and vector management strategies in central Pennsylvania.

## Highlights

• MG had higher specificity and sensitivity in detecting tick-borne pathogens over PCR.
• Relationships among tickborne pathogens were elucidated.
• Significant multivariate associations found between metadata and microbial species.

## Graphical abstract

**Figure:**
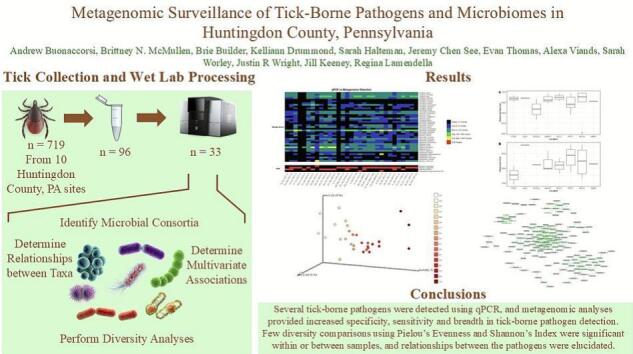

## 1 Introduction

Tick populations have grown rapidly in the past few decades, globally increasing the prevalence of tick-borne illnesses transmitted due to the various pathogens ticks carry [1]. During a 15-year period from 1992 to 2006, 248,074 total cases of the tick-borne illness Lyme disease was reported in the United States, increasing from 9908 cases in 1992 to 19,931 cases in 2006 (101 % annual growth rate) [2]. From 2019 to 2022, there were 184,459 (on average, about 46,000 annually) reported cases of tick-borne illnesses in the United States, though that number is thought to be biased due to the Covid-19 pandemic [3]. Binghamton University's Tick-borne Disease Center now claims about 500,000 new cases of tick-borne diseases occur annually [4]. Of all the vector-borne diseases found in the United States, 95 % are transmitted by ticks [5].

Ticks exhibit highly localized adaptations to warm, humid abiotic conditions and specific host species, which have historically restricted the ranges they can inhabit [6]. However, ecological shifts have resulted in expanded ranges of known tick species [7,8] and the past two decades have seen a rise in novel tick-borne pathogens identified in the USA [9]. For instance, reforestation efforts in the 20th century have expanded the once rare *Ixodes scapularis* tick, whom is now responsible for the largest share of vector-borne illnesses in North America, across the eastern U.S. and southeastern Canada [6,[10], [11], [12]]. Rising temperatures have also facilitated the spread of *I. scapularis,* as the species is highly sensitive to desiccation and cannot survive extremely low temperatures (< − 10 °C) for prolonged periods [7,8,13].

Advancements in molecular techniques have significantly enhanced the detection and cataloging of tick-borne pathogens in recent years [14,15]. Longstanding morphology techniques such as microscopy and cultures were hindered by low pathogen load, high taxonomic diversity, and difficulty distinguishing closely related tick-borne pathogens [14]. Serology-based tests have been the gold standard in detecting antibodies of tick-borne pathogens, usually starting with a two-step enzyme-linked immunosorbent assay (ELISAs) followed by western blotting if the ELISA was positive [14,16]. ELISA and western blot accuracy, however, varies widely. For example, when testing Lyme borreliosis in Europe, they have been shown to have an average sensitivity of only about 80 % and a specificity of about 95 %, leading to false positives and negatives [16]. Polymerase Chain Reaction (PCR) offers a different approach by directly amplifying even low concentrations of a pathogen's genetic material to identify tick-borne pathogens. As a result, PCR can detect rare or emerging variants that might otherwise be overlooked and has the potential to alter the classification of species, as demonstrated most saliently by the reclassification of *Anaplasma phagocytophilum* in 2001 [15].

While PCR marked a significant step forward in the detection and classification of tick-borne pathogens, the emergence of highly parallelized sequencing technologies has revolutionized the field by enabling direct sequencing of total nucleic acids. Metagenomics, which involves sequencing all genomes present in an environmental sample, is widely used to investigate diverse microbial communities [17]. This approach can be implemented through targeted amplicon sequencing or untargeted shotgun methods [18]. Although shotgun metagenomics is typically slower and more costly than targeted approaches, it offers the distinct advantage of detecting a greater array of organisms present in a sample, including unexpected or novel pathogens [14]. With the rising prevalence and public health impact of emerging tick-borne pathogens, shotgun metagenomics is an increasingly valuable tool for comprehensive pathogen surveillance and discovery.

As the range and overall abundance of tick-borne pathogens continue to grow, it is critical to understand the diversity and geographic distribution of tick species at the local level. This knowledge is essential for informing public health policies aimed at reducing exposure to disease vectors and improving the diagnosis and treatment of tick-borne illnesses. Current diagnostic methods are primarily limited to targeted testing for known pathogens, leaving a substantial gap in our ability to detect novel or unexpected agents of disease [19]. Without comprehensive surveillance, potentially significant tick-borne pathogens may go undetected, hindering effective clinical response and public health preparedness.

From 2019 to 2022, Pennsylvania accounted for 14.4 % of tick-borne disease nationally, including 23,645 cases of Lyme disease, the second-most of any state during that period [3]. The rising number of disease cases has caused a sustained increase in tick-bite related emergency visits throughout all regions of the state [20]. Huntingdon County, Pennsylvania, is an excellent setting for intensive surveillance of tick populations due to its rural location and the popularity of outdoor recreational activities in the county that bring the threat of ticks and their associated illnesses. The county hosts forests, hiking trails, state parks, and Raystown Lake, which attracts locals year-round and many visitors seasonally. These outdoor locations are also home to a large number and variety of ticks, including but not limited to, *Ixodes scapularis* (Blacklegged or Deer Tick), *Dermacentor variabilis* (American Dog tick), *Amblyomma americanum* (Lone star tick), *Haemaphysalis longicornis* (Asian Longhorned tick), and *Amblyomma maculatum* (Gulf Coast tick) [21]. Accordingly, the residents of, and visitors to, Huntingdon County, PA are at significant risk of tick-borne pathogen exposure and subsequent illness. The county rate of reported tick-borne illness per capita is over six times the national average [3]. Surveillance of ticks and their respective pathogens is imperative to the county's public health initiatives.

In this study, we investigate the distribution and abundance of tick species across Huntingdon County in the summer of 2023 by systematically sampling from a range of heavily trafficked outdoor recreational sites. Using both PCR and shotgun metagenomics, we analyze the microbial communities present in individual tick specimens to catalog the diversity of tick-borne pathogens comprehensively. This integrative approach enables the identification of high-risk areas and patterns of pathogen prevalence, generating actionable data to inform local public health strategies, guide mitigation efforts, and enhance community outreach initiatives aimed at reducing tick-borne disease transmission.

## 2 Materials and methods

### 2.1 Sample collection using the drag cloth method

#### 2.1.1 Materials

To begin sample collection, a measuring wheel, a minimum of 10 flags, drag cloths, at least 50 Eppendorf tubes filled with 50 mL 75 % or higher ethanol, fine-tipped tweezers, plastic bags to hold tubes with samples, a blank data collection sheet, a densiometer, a thermohygrometer, and a GPS are needed.

#### 2.1.2 Construction of dragnet cloths

A 114 cm by 102.4 cm piece of white flannel was cut. The 102.4 cm side was hemmed by folding over 1.2 cm from each edge and sewing it down. A loop of fabric was created at the top, and a pocket was created at the bottom of the 114 cm length side to hold the dowel by folding and sewing 7 cm on each side. One end of the bottom pocket is sewn shut. The total area of the entire square cloth is therefore 1 m^2^. A 1-m-long, heavy, metal chain is inserted into the bottom pocket and pinned into place using large baby pins. A wooden dowel rod, approximately 1.143 m long, is inserted through the top loop. Two holes were drilled through the dowel 3.8 cm from each end. A 3 m rope is fastened through the holes of the dowel rod, which creates a handle from which the net can be pulled. Three total dragnets were constructed [22]. This design ensures efficient deconstruction and reconstruction of the dragnets so the 1 m^2^ flannel can be bleached and washed as needed.

#### 2.1.3 Sample and environmental data collection

Sample collection was conducted between May and August to coincide with the peak seasonal activity of adult ticks [23]. The CDC's approved drag-netting protocol for tick collection is divided into two steps: flagging and dragging [24]. At every collection site, data is collected at ten, 75-m-long transects for a total, standardized sample area of 750 m [22]. Each transect covers a 1-m by 75-m area. The start of each transect is found using a measuring wheel to go a predetermined distance along the trail. This predetermined distance is calculated by dividing the total length of the trail by subtracting 750 m from the total trail length, then dividing by 10 to ensure even sample collection coverage of the trail. Each transect is split into five 15-m sections, also called stops, where a flag is placed at the end of each stop.

To begin flagging, the measuring wheel is reset to read zero. Wheel the measuring tape along the floor of the trail until the predetermined distance is reached. A flag is placed to indicate the start of a transect. 15 m is measured in a direction where it will be possible to drag, and a flag is placed at the end of this. This is repeated four more times so that a flag marks the beginning and end of each stop within the transect. A total of 6 flags are placed during this process.

Dragging is completed on each transect using a clean dragnet. The drag cloth is dropped at the first flag, indicating the beginning of the transect. The dragnet is pulled until the end of the stop, which is indicated by the next flag. The dragnet is inspected for ticks. If any samples are found, they are systematically removed using tweezers and placed in an Eppendorf tube filled with between 0.25 mL and 0.75 mL 95 % ethanol to prevent DNA degradation and loss of data. Each sample collection tube is labeled to indicate which transect and stop they were collected at. The transects are coded using A to J, and the stops are coded as 1–5 for each transect. If multiple samples are found at one stop, they are all placed in the same tube, and the number of ticks collected is labeled on the tube. For example, a tube containing 7 tick samples collected at the third stop of the 9th transect would be labeled I.3.7.

Environmental data is collected at the beginning of each stop on each transect. GPS coordinates are gathered using a GPS, and a densiometer determines canopy cover. Further, a thermohygrometer measured the temperature and humidity of the location.

**Fig. 1:**
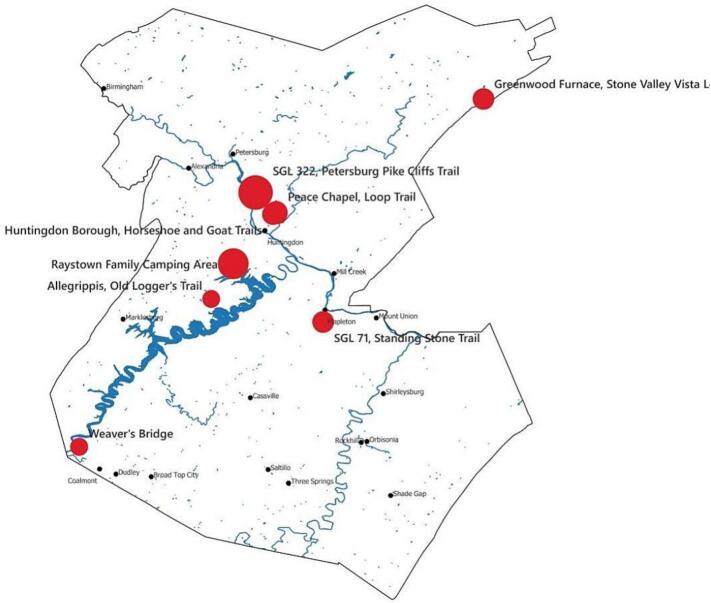
Map of Huntingdon County, Pennsylvania. The locations of the sites sampled across Huntingdon County are labeled and marked with red dots with radii proportional to the number of ticks from each site that tested positive for at least one pathogen in our PCR screening. No pathogens were detected by PCR at SGL 81 or Trough Creek, and none of the ticks found on those sites were sequenced, so they were omitted from the diagram. Population centers are labeled and marked with black dots, and waterways are shown in blue. (For interpretation of the references to colour in this figure legend, the reader is referred to the web version of this article.)

Samples that have already been processed were collected from high-trafficked trails between 6/5/2023 and 7/31/2023 during the summer of 2023 and between 5/29/24 and 6/19/2024 during the summer of 2024, at 10 high-use recreational areas in Huntingdon County. These were the Peace Chapel Loop Trail, Allegrippis Old Loggers Trail, Petersburg Pike Cliff Trail, Raystown Family Camping area, Trough Creek, Greenwood Furnace Stone Valley Vista Loop, Weaver's Bridge, Horseshoe and Goat Trails, SGL 81 Standing Stone Trail, and SGL 71 Standing Stone Trail (Fig. 1). In total, each trail was sampled twice except Peace Chapel Goat Trail, Peace Chapel Loop Trail, and Weaver's Bridge, where samples were collected three total times. Huntingdon County community members also submitted ticks collected from household pets.

### 2.2 DNA/RNA extraction and PCR testing for tick-borne pathogens

All tick samples were sent to the Dr. Jane Huffman Wildlife Genetics Institute at East Stroudsburg University for tick-borne pathogen testing using DNA/RNA extractions and qPCR. The selected pathogens were screened for molecular detection using qPCR; species-specific testing for *Borreliella burgdorferi* (Lyme disease), *Anaplasma phagocytophilum* (Anaplasmosis) including the distinction of the two variants (human and deer variants), *Babesia microti* (Babesiosis), and *Borrelia miyamotoi* (Hard Tick-borne Relapsing fever), and genus-specific testing for *Borrelia* species (spp) and *Babesia* species (spp).

Ticks were processed at a single-tick resolution, starting with midsagittal cuts with sterile scalpels, followed by DNA/RNA extraction (additional details in the following paragraph). Blank controls were included with every 30 samples, with an additional blank for the remaining six samples, PCR negative controls, and an internal positive control for Ixodidae were used to validate both nucleic acid extraction and PCR protocols, and to ensure the absence of contamination. A species-specific TaqMan multiplex assay, SYBR duplex assay (*Borrelia* spp. and *Babesia* spp.), was used and validated at the Dr. Jane Huffman Wildlife Genetics Institute [25].

Microscopy of each tick sample collected is used to identify size, life stage, and species using the Tick Identification Guide from the Tick Research Lab of Pennsylvania [26]. After this, the remaining tick samples not sent for external analysis are processed using DNA extraction, traditional nPCR, and gel electrophoresis. DNA was extracted from tick samples using the Zymo “Quick-DNA Pathogen MagBead” kit. Minor changes were made to the standard protocol. After removing the ticks from the ethanol, they were dried completely before adding them to a nuclease-free tube with 400 μL DNA Shield. Ticks were mechanically crushed using tweezers for about 3 min per sample. Samples were placed in the Disruptor Genie for 15 min, instead of 10, after being transferred to the MagBead lysis tube. Instead of incubating the samples in the Proteinase K treatment for only 15 min, 24–48 h resulted in higher concentrations of extracted DNA. Further, the DNase I treatment was not utilized. 30 μL DNase-free water was used to elute the DNA, but the exact concentration of the final DNA extractions was not determined.

Nested PCR was used to amplify and ID the *Borrelia burgdorferi* flagella (FlaB) gene and *Ixodes scapularis* actin gene. The nPCR procedure and primer sets for *B. burgdorferi* identification were adapted from Wills et al. in “Detecting the Lyme Disease Spirochete, *Borrelia burgdorferi*, in Ticks Using Nested PCR” [27]. In addition to the use of the FlaB primer sets, an actin primer set was developed by utilizing BLAST alignment of the *Ixodes scapularis* actin gene, *Canis lupus familiaris* actin gamma 1, *Homo sapiens* actin alpha 1, skeletal muscle (ACTA1), and *Felis catus* actin beta (ACTB), selecting a region of least homology between these organisms.

Primers were ordered from IDT (Integrated DNA Technologies) in Coralville, IA. These actin primers were validated by testing on an *I. scapularis* DNA extraction as a positive control. They did not work on PCR amplification of human, cat or dog DNA. The specific primer sets, target sequences, amplicon sizes, and annealing temperatures are listed in Table 1.

**Table 1:** List of primers and their target sequences used in the nPCR protocol.

Primer Name	Gene	Target Sequence (5′ → 3′)	Amplicon Size (bp)	Annealing Temperature (°C)
FlaB Out Fw	FlaB	gcatcactttcagggtctca	503	55
FlaB Out Rv	FlaB	tggggaacttgattagcctg		
FlaB In Fw	FlaB	ctttaagagttcatgttggag	447	58
FlaB In Rv	FlaB	tcattgccattgcagattgt		
Actin Fw	Actin	ccatcctccgtctggact	200	56.1–56.3
Actin Rv	Actin	gtcgggaagctcgtagga		

The thermocycler program settings used are indicated in Table 2 and were adapted from Wills et al. [27].

**Table 2:** Thermocycler settings for nPCR of *Ixodes scapularis* actin and *B. burgdorferi* FlaB.

*B. burgdorferi* FlaB Nested PCR1 and *I. scapularis* actin PCR (containing		*B. burgdorferi* FlaB Nested PCR2	
BURG.N.1	(40 cycles)	BURG.N.2	(40 cycles)
95 °F (denature)	5’	95 °F (denature)	5’
95 °F (denature)	15″	95 °F (denature)	15″
55 °F (anneal)	30″	58 °F (anneal)	30”
68 °F (extend)	45”	68 °F (extend)	45”
68 °F (extend)	5’	68 °F (extend)	5’
4 °F	Forever	4 °F	Forever

The actin primer set is only necessary in the first reaction of nested PCR. In the second round of nPCR, the inner FlaB forward and reverse primers are utilized at the concentrations and volumes listed in Table 3.

**Table 3:** Initial PCR and nested PCR.

Reagents	Volumes for Initial PCR	Volumes for Nested PCR
2× Taq mix (NEB)	12.5 μL	12.5 μL
FlaB FW & RV primers (10 μM)	1 μL outer primers	1 μL inner primers
Actin FW & RV (10 μM)	1 μL	
DNA Template	2 μL from sample extraction	2 μL from initial PCR
RNAse-Free Water	6.5 μL	8.5 μL
**Total**	**25 μL**	**25 μL**

Following completion of the nPCR reactions, agarose gel electrophoresis (2 % final, 0.887 M Tris, 0.284 M Taurine, and 6.33 mM EDTA buffer) is done to visualize the DNA. The gels were run at 80 V for 50 min before being visualized under UV light.

### 2.3 Metagenomics library preparation and sequencing

Wright Labs, LLC received residual DNA extracts from the Dr. Jane Huffman Wildlife Genetics Institute at East Stroudsburg University. Concentrations for all extracts were quantified using an Invitrogen Qubit Flex Fluorometer and 1× Qubit dsDNA High Sensitivity Assay Kit (ThermoFisher Scientific, Waltham, MA). All DNA extracts except for two with sufficient concentration for the library preparation kit were concentrated using a vacufuge (Vacufuge Plus, Eppendorf, Hamburg, Germany) for 50 min to result in an approximate remaining volume of 35 uL. Concentrated extracts were again quantified using the Qubit Flex Fluorometer and 1× Qubit dsDNA High Sensitivity Assay Kit (Thermo Fisher Scientific, Waltham, Massachusetts).

Samples that tested positive for at least one pathogen in PCR were selected for metagenomics (33 of the 96 samples), where their quantified DNA extracts were used as normalized input for the Nextera XT DNA Library Preparation Kit (Illumina, San Diego, CA). Libraries were quality checked using an Agilent 2100 Bioanalyzer and DNA High Sensitivity kit (Agilent Technologies, Santa Clara, CA) and then pooled in an equimolar ratio. The pool was gel-purified using a 2 % agarose gel and the Qiagen QIAquick gel extraction kit (Qiagen, Germantown, MD, USA). Following purification, the pool was stored at −20 °C and was then sequenced on an Aviti sequencing platform (Element Biosciences, San Diego, CA, USA) to produce 2 × 150 reads.

### 2.4 Bioinformatics analysis of metagenome data

After sequencing, FastQC [28] reported raw data quality. Fastp [29] was used to filter reads based on the reported quality using a sliding window of 4 bp with a minimum average quality of 28 and minimum length of 90 bp. The Kraken2 program [30] then assigned National Center for Biotechnology Information (NCBI) taxonomic identifiers to the samples' sequences and generated taxonomy reports that were later used to create a table for species-level analysis. *Homo sapiens* were removed via the SeqKit grep command based on assigned taxonomic ids [31] before functional annotation to avoid human contamination from influencing analysis.

Sequence quality was further improved using the PEAR (pair-end read merger) program [32] to merge the forward and reverse reads for each sequence. EggNOG-mapper [33] then used the EggNOG database [34] to carry out functional annotations for each sequence. Results were collated in tables that listed the Kyoto Encyclopedia of Genes and Genomes (KEGG) Orthologs (KOs) abundance per sample and the taxa contributing to each KO [35].

Both Kraken2 and EggNOG-generated data were used for downstream analysis, which was performed using a QIIME2–2022.11 [36] and R-centric pipeline [37]. Initial Kraken2 was completed using a modification of its “standard” database that also included fungi (in addition to the archaea, bacteria, human, plasmid, viral, and UniVec_Core that are normally included libraries) database to identify reads [38]. That database encompasses a wide range of microbial taxa, but crucially lacks *Babesia microti* and *Babesia divergens,* along with other eukaryotic pathogens. Since 12 samples tested positive for *Babesia spp* in our PCR screening, we ran Kraken2 again with the k2_eupathdb48_20230407 database, which includes only eukaryotic pathogens, including many species of the *Babesia* genus. We merged the species counts tables from the original Kraken2 analysis and the secondary eukaryotic follow-up for all further analysis so that potential relationships between eukaryotic pathogens and other microbiomic features could be examined.

### 2.5 Statistical analysis

Downstream statistical analysis was performed on metadata collected on the day of tick collection to analyze the parameters under which the ticks were found. Ticks were compared by the following discrete metrics: the date, month, location, land cover, and time of day the samples were collected, and PCR results for the common tick pathogens *Borreliella burgdorferi*, *Anaplasma phagocytophilum* (human variant), *Borrelia miyamotoi*, *Babesia* species, and *Borrelia* species. Continuous variables were also metrics of comparison and included: the number of ticks detected, canopy cover squares, percentage of canopy cover, temperature (°C), and percent humidity at the time of tick collection.

Resulting plots were visualized in qiime2–2022.11 or R. Alpha diversity was calculated for each comparison using the metrics Observed Features, Pielou's Evenness, and Shannon's Index based on average values at a rarefaction depth of 14,400 to more easily compare across cohorts (even if samples had different sequencing depths). Kruskal-Wallis tests (overall and pairwise) were performed for categorical metadata, and alpha diversity values were visualized as boxplots.

Beta diversity analysis was also conducted for each comparison for both microbial taxa and KO datasets after CPM normalization and visualized with PCoA plots. PERMANOVA tests were performed on generated Bray-Curtis dissimilarity matrices to observe differences between categorical metadata samples. Mantel tests were performed on continuous metadata to see if samples differed significantly based on metadata cohorts. Adonis was also performed on continuous metadata to elucidate how much variation could be explained by continuous metadata.

LefSe [39] plots were created based on categorical metadata with CPM normalized data to display differential taxa. MaAsLin2 [40] determined multivariate associations between metadata cohorts and microbial meta-omics features, such genes, pathways, and species.

A co-occurrence network was created using Kraken2 species-level annotations. The network files were produced using CoNet [41]. The edge parameter was set to 500, and a minimum occurrence of five samples was required to be considered. Correlations were calculated using Spearman's rank correlation with a threshold of 0.05 and a minimum *p*-value of 0.05. The network visualization was created using the CoNet app on Cytoscape version 3.8.2 [42]. The nodes were organized using CoSe spring embedder.

## 3 Results

### 3.1 Initial pathogen screening

**Fig. 2:**
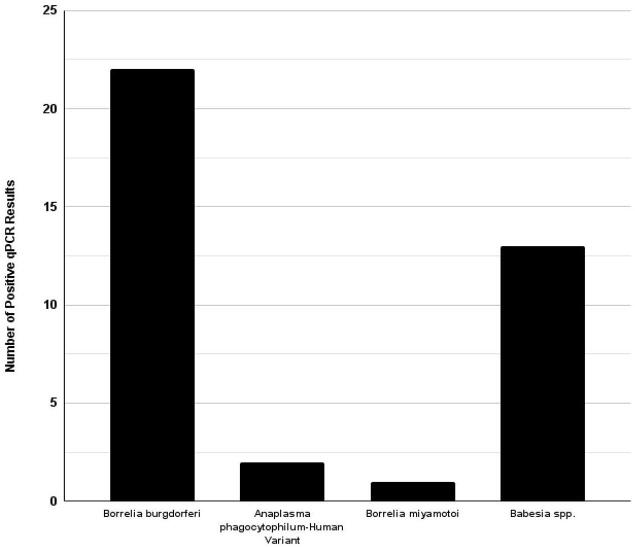
Bar graph of PCR results. The makeup of positive PCR results by pathogen across all sites were visualized as a bar graph.

All 96 samples initially tested were *Ixodes scapularis* ticks, and included 84 adults, 2 nymphs, and 10 larvae. Of the 33 sampled ticks for metagenomic analysis, several screened pathogens were identified (Fig. 2). A total of 22 of the 96 ticks tested positive for *Borreliella burgdorferi* and one tick was positive for *Borrelia miyamotoi*. Thirteen of the 96 ticks tested positive for *Babesia* spp. Two of the 96 ticks were positive for *Anaplasma phagocytophilum* (human variant). None of the samples tested positive for *Babesia microti* or *Anaplasma phagocytophilum* (deer variant). Additionally, there were five co-infections (5.21 %) identified via PCR. Four co-infections were between *Borreliella burgdorferi* and *Babesia* spp. The fifth co-infection was between *Borreliella burgdorferi* and *Anaplasma phagocytophilum* (human variant).

### 3.2 General sequencing counts

Metagenomic analysis of ticks which tested positive for at least one pathogen in our qPCR screening was completed using Kraken2. In total, 6,389,322 reads representing 9924 different microbial taxa were identified across both sequencing runs. Additionally, 8129 different KEGG orthologs were detected with a total summed reads-per-kilobase normalized total of 1,122,270.637. Two of the 33 PCR-screened samples were excluded from downstream analysis past this point due to absent metadata.

### 3.3 PCR vs. metagenomic pathogen detection

**Fig. 3:**
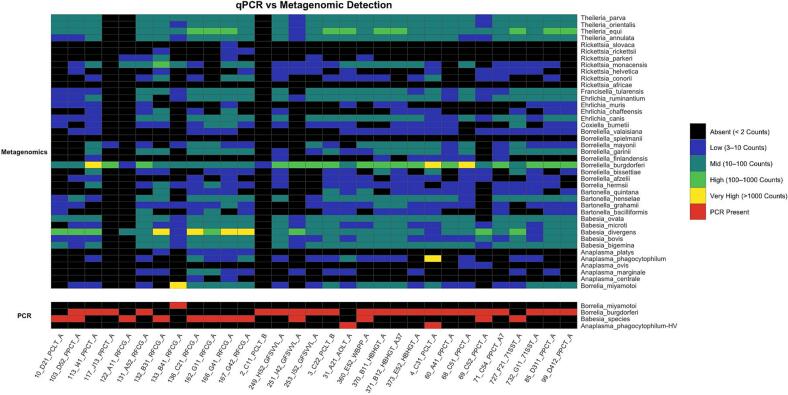
Presence absence heatmap comparing qPCR detection and metagenomic detection (binned counts) of tick-associated pathogens across tick samples (*n* = 31). Each row represents a tick-associated pathogen, and each column represents an individual tick sample. qPCR detection is shown in red (presence) or black (absence). Metagenomic detection is displayed as a binned colour scale: black (absent, <2 counts), blue (low, 3–10 counts), green (mid, 10–100 counts), yellow (high, 100–1000 counts), and bright yellow (very high, >1000 counts). This figure provides an overview of detection concordance between methods, while also highlighting additional pathogens detected exclusively by metagenomics. The inclusion of metagenomic sequencing enables broader pathogen detection beyond targeted qPCR panels, offering improved insight into pathogen diversity and abundance across collected tick samples. (For interpretation of the references to colour in this figure legend, the reader is referred to the web version of this article.)

Comparison of qPCR and shotgun metagenomic data revealed notable differences in both the breadth and sensitivity of pathogen detection (Fig. 3). Shotgun metagenomics outperformed PCR in both sensitivity and breadth of detection across the 31 tick samples analyzed. While PCR detected a total of 46 pathogen occurrences limited to four taxa: *Borreliella burgdorferi*, *Babesia divergens*, *Borrelia miyamotoi*, and *Anaplasma phagocytophilum*, metagenomics identified 63 occurrences of these same taxa. Furthermore, metagenomics revealed co-infections in 22 out of 31 samples, notably identifying the concurrent presence of *Babesia divergens*, *Borreliella afzeli*i, and various *Rickettsia* species in multiple cases. It also identified pathogens in samples that PCR classified as negative (*n* = 63), indicating increased detection sensitivity. These results underscore the broader taxonomic resolution and higher potential sensitivity of shotgun metagenomics compared to conventional PCR.

Concordance between PCR and metagenomics for the four shared targets ranged from 74 % to 94 %. The highest agreement was observed for *Borrelia miyamotoi* (94 %), indicating strong consistency in platform detection. Slightly lower concordance was found for *Babesia divergens* (90 %) and *Borreliella burgdorferi* (87 %). Conversely, there were only four ticks that were observed where PCR was positive, but metagenomics showed low (≤ 10 reads) or absent read counts, likely due to sampling variation, sequencing depth, or taxonomic assignment limitations. The lowest concordance was noted for *Anaplasma phagocytophilum* (74 %), indicating more frequent discrepancies between the two methods.

### 3.4 Alpha diversity

**Fig. 4:**
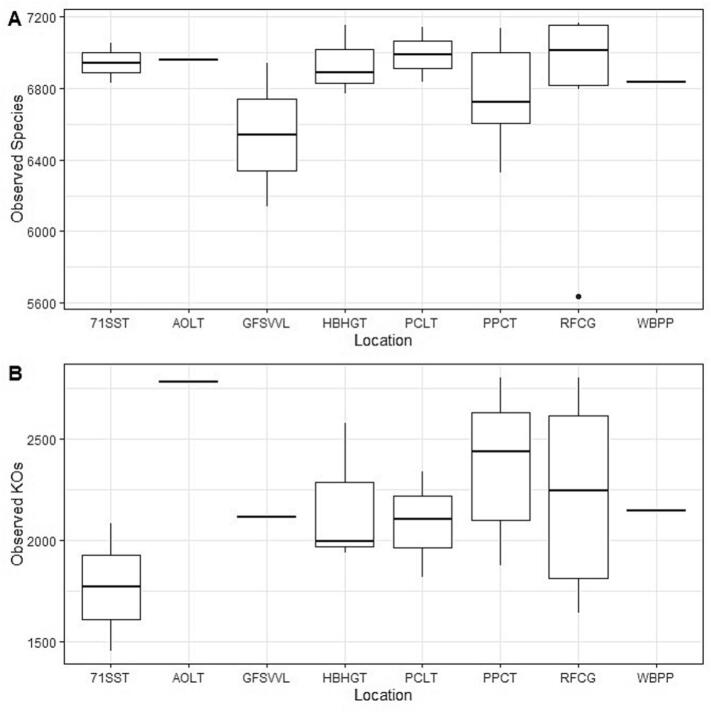
Boxplots of alpha diversity (observed features) by location, comparing (A) taxonomic diversity and (B) gene diversity. Panel A displays species-level richness across sampling locations, while Panel B illustrates diversity based on predicted functional gene expression profiles. Each boxplot represents the distribution of observed features per location. The analysis was performed using QIIME 2 (version 2022.11) and visualized via view.qiime2.org.

Statistical analysis comparing alpha diversity within tick samples to the metadata features date, month, location, land cover, time of day, number of ticks collected, canopy cover squares, percent canopy cover, temperature, and percent humidity at the time of sample collection revealed no overall significant differences in microbial species or identified genes when considering the alpha diversity metrics observed features, Pielou's Evenness, and Shannon's Index. While no overall significant differences were elucidated, a range of alpha diversity values were revealed. For example, the number of observed features when considering species and location ranged from about 6150 to 7150 observed features (Fig. 4A) and the number of observed features when considering identified genes and location ranged from 1450 to 2810 observed features (Fig. 4B).

Comparisons of microbial species showed that no metadata category corresponded to significant differences in alpha diversity values (*p* > 0.05). However, the number of ticks detected was significantly correlated with Pielou's evenness (Spearman's ρ = 0.4168, *p* = 0.0479) and Shannon's index (Spearman's ρ = 0.4142, *p* = 0.0494), where the microbial evenness and richness within each tick sample increased as the number of ticks detected increased.

Comparing identified genes revealed no significant differences in continuous metadata but revealed several values of significance in discrete metadata (Table 4,SMFIG 2). The month of June had higher alpha diversity than July (SMFIG 2 A), which was generally consistent with pairwise comparisons of dates. June 15, 2023 had the highest alpha diversity of all dates considered and July 13, 2023 had the lowest (SMFIG 2B). Pairwise comparisons in location were also significant, where PPCT had the highest alpha diversity and 71SST had the lowest. The alpha diversities of HBHGT and RFCG were closer to the median and the interquartile ranges of their boxplots were larger than those of PPCT and 71SST (SMFIG 2C). Lastly, the early afternoon time of day had a higher alpha diversity than the late afternoon (SMFIG 2D).

**Table 4:** Alpha diversity metrics comparing month, date, location, and time of day to identified genes.

Metadata Variable	Alpha Metric	Cohort 1	Cohort 2	H	p-value	q-value
Month	Shannon's Index	July (*n* = 7)	June (*n* = 17)	3.7523	0.0527*	0.0527*
	Pielou's Evenness	July (n = 7)	June (n = 17)	4.5267	0.03334*	0.0334*
Date	Shannon's Index	6/15/2023 (*n* = 6)	6/21/2023 (n = 7)	6.6122	0.0101*	0.2836
			7/26/2023 (*n* = 2)	4.0000	0.0455*	0.5226
	Pielou's Evenness	6/15/2023 (n = 6)	6/21/2023 (n = 7)	5.2245	0.0223*	0.4247
			7/13/2023 (n = 3)	4.2667	0.0389*	0.4247
			7/26/2023 (n = 2)	4.0000	0.0455*	0.4247
Location	Shannon's Index	71SST (n = 2)	PPCT (n = 6)	4.0000	0.0455*	0.5226
		PPCT (n = 6)	RFCG (n = 7)	6.6122	0.0101*	0.2836
	Pielou's Evenness	71SST (n = 2)	PPCT (n = 6)	4.0000	0.0455*	0.4247
		HBHGT (n = 3)	PPCT (n = 6)	4.2667	0.0389*	0.4247
		PPCT (n = 6)	RFCG (n = 7)	5.2245	0.0223*	0.4247
Time of Day	Pielou's Evenness	Early Afternoon (*n* = 8)	Late Afternoon (*n* = 5)	4.8214	0.0281*	0.0843

Alpha diversity statistics were calculated using the alpha diversity metrics Pielou's Evenness and Shannon's Index, though observed features were also considered. Only comparisons with a p ≤ 0.05 were included. Both p-values and q-values ≤0.05 were designated with an asterisk (*).

### 3.5 Beta diversity

Statistical analysis comparing beta diversity among tick samples to the metadata features previously mentioned revealed no overall significant differences in microbial species or identified genes. PERMANOVA (overall and pairwise) values and the Mantel Test were considered.

**Fig. 5:**
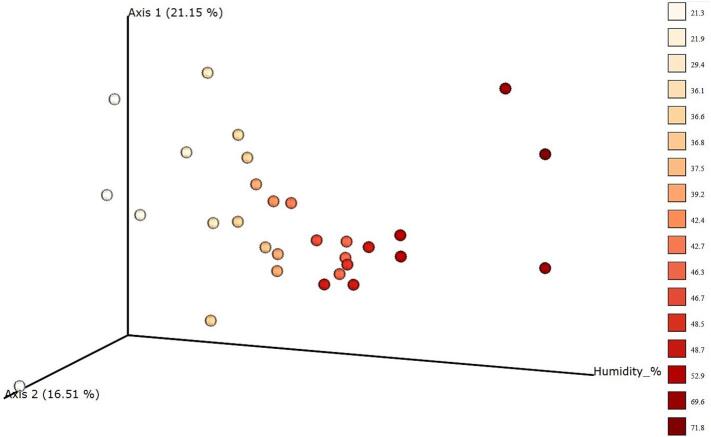
Principle Coordinate Analysis (PCoA) plots visualizing phylogenetic similarities between samples of varying percent humidities. Mantel tests and Adonis were performed on continuous metadata to see if samples differed significantly based on metadata cohorts and to elucidate how much variation could be explained by continuous metadata, respectively. PCoA plots were visualized in view.qiime2.org.

Comparing microbial species revealed no discrete metadata resulted in significant beta diversity values (*p* > 0.05), but percent humidity (PERMANOVA, pseudo-F = 0.2309, *p* = 0.039) featured significant differences in the phylogenetic relationships between samples (Fig. 5). Mid-to-high percent humidities appeared to cluster more tightly than lower and the maximum percent humidities observed.

Comparisons of identified genes revealed similar findings, where only land cover (PERMANOVA, pseudo-F = 1.9274, *p* = 0.028) and time of day (PERMANOVA, pseudo-F = 1.5822, *p* = 0.042) revealed significantly different phylogenetic relationships. Driving those overall differences were the pairwise comparisons of leaf litter and short grass (PERMANOVA, pseudo-F = 2.970808, p = 0.028, q = 0.084) for land cover, and for time of day, early and late afternoon (PERMANOVA, pseudo-F = 1.8741, *p* = 0.052, q = 0.078) and early afternoon and late morning (PERMANOVA, pseudo-F = 1.9573, *p* = 0.024, q = 0.072).

### 3.6 Multivariate associations between collected metadata and identified microbial species

Multivariate associations were elucidated. Considering the variable Land Cover, the taxon *Leptomonas seymouri* was enriched in the Leaf Litter cohort (MaAsLin2, negative coefficient, q = 0.04211768701) when compared to Tall Grass (Table 5).

**Table 5:** MaAsLin2 determined multivariate associations between metadata and microbial taxa.

Microbial Sepcies	Metadata Variable	Cohort	Effect Size	stderr	N	N.not.0	p-value	q-value
*Leptomonas seymouri*	Land Cover	Tall Grass	−0.7522	0.152	30	30	3.20E-05	0.0421
*Borreliella burgdorferi*	*Borrelia* species	POS	3.1413	0.5644	31	31	5.28E-06	0.007
*Borreliella burgdorferi*	*Borreliella* burgdorferi	POS	3.3376	0.4869	31	31	1.57E-07	0.0002
*Babesia divergens*	*Babesia* species	POS	5.2433	0.5649	31	30	3.49E-10	4.59E-07

This table reflects only multivariate associations with a significant q-value (q < 0.05) for the respective metadata and microbial taxa identified at the species level. Associations of features enriched in Tall Grass were denoted with a positive coefficient and those enriched in Leaf Litter were denoted with a negative coefficient for Land Cover. Associations of features enriched in samples that tested positive for *Borrelia* species, *Borreliella burgdorferi*, and *Babesia* species were denoted with positive coefficients, while those enriched in samples that tested negative for *Borrelia* species, *Borreliella burgdorferi*, and *Babesia* species were denoted with a negative coefficient.

Samples that tested PCR positive for the common tick pathogens *Borrelia* (any species), *Borreliella burgdorferi*, and *Babseia* were enriched with two, one, and one taxa (MaAsLin2, positive correlation, q < 0.05), respectively, compared to tick samples that did not test positive for those common tick pathogens (MaAsLin2, negative correlation, no q < 0.05). There were no taxa enriched (q > 0.05) whether the tick samples tested positive or negative for the common tick pathogen *Anaplasma phagocytophilum* (human variant)*.*

### 3.7 Network analysis of microbial associations in the tick microbiome

**Fig. 6:**
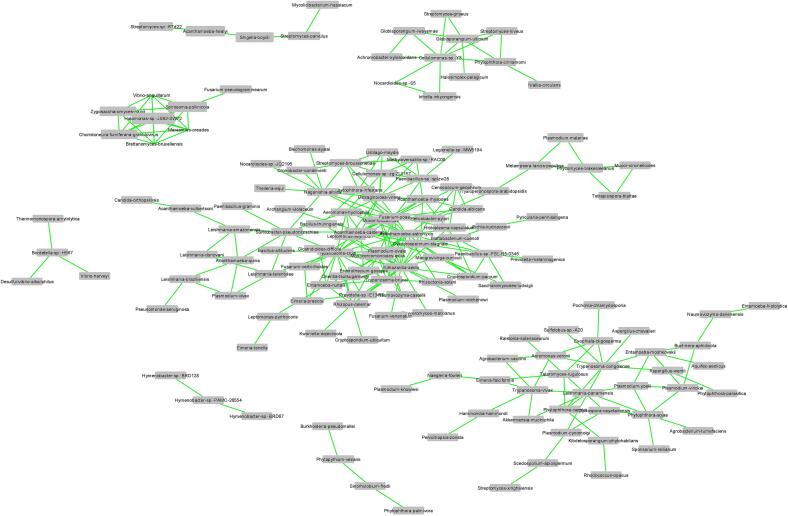
Co-occurrence network. Nodes are labeled by species name and sized by relative abundance. Edges indicating positive associations between the connected nodes are shown in green, and those indicating negative associations are shown in red. Associations were measured by pairwise Spearman Correlation with minimum threshold of 0.05 and *p*-values <0.05. Pairs of nodes that were only connected to each other were omitted from the final network. We omitted taxa which occurred in less than 5 samples.

The co-occurrence network (Fig. 6) depicts microbial species interactions based on pairwise correlations, with nodes representing species and edge colors indicating correlation direction (green for positive, red for negative). In this network, positive associations dominate, suggesting widespread mutualism or shared ecological niches among taxa in the tick microbiome. The central cluster shows high connectivity, with a dense core of species such as *Lactobacillus*, *Bacteroides*, and *Prevotella* serving as network hubs, indicating their potential role in structuring the tick-associated microbial community. Several peripheral subnetworks also emerged, including tightly connected hubs that may represent functionally or phylogenetically related taxa. Notably, no negative correlations were observed, indicating that only positive correlations were retained or prevalent above the correlation threshold of 0.05. Low-abundance taxa were omitted, improving visual clarity and network interpretability. Upon inspection, *Rickettsia* was the only known tick-borne pathogen genus present in the network. Positioned centrally within a dense cluster, *Rickettsia* exhibited multiple positive associations with other taxa, suggesting frequent co-occurrence and a dominant role in the microbiome. In contrast, *Borrelia*, *Anaplasma*, *Ehrlichia*, and *Babesia* were not detected in the network. Their absence may reflect low abundance, limited distribution across samples, or exclusion based on filtering criteria.

## 4 Discussion

Overall, several tick-borne pathogens were detected using qPCR, and metagenomic analyses provided increased specificity, sensitivity and breadth in tick-borne pathogen detection. Few diversity comparisons using Pielou's Evenness and Shannon's Index were significant within or between samples, and relationships between the pathogens were elucidated.

### 4.1 Pathogen screening

Of the screened tick-borne pathogens, *Borreliella burgdorferi* tested positive in the most ticks (23.40 %). *Borreliella burgdorferi*'s primary vector is the black-legged tick (*I. scapularis*), which parasitizes small mammals and birds. The pathogen causes Lyme disease in humans, the most common vector-borne disease in North America, and presents with a characteristic annular rash, arthritis, carditis, and in late stages, encephalopathy [43,44]. Though antibiotic treatment is available, many patients live with persistent symptoms six months post treatment and are diagnosed with post-Lyme disease syndrome [45]. The commonplace occurrence of *Borreliella burgdorferi* along with the significant long-term health complications posed by Lyme disease make surveillance and awareness of the pathogen imperative in central PA.

PCR screening detected *Borrelia miyamotoi*, the causative agent of *Borrelia miyamotoi* disease (BMD), in one sample in this study [46]. While less common than Lyme disease, BMD presents a range of symptoms (anywhere from flu-like symptoms to cognitive and physical difficulties) that highlight the importance of tracking this pathogen [46]. *B. miyamotoi* has been detected in *I. pacificus* and *I. scapularis*, which is the same hard tick species that can transmit *Borreliella burgdorferi* in both the nymph and adult stage of the ticks life [46].

*Babesia* spp. was well represented in these samples, being found positive in 13.83 % of them. *Babesia* are vectored by hard ticks (notably from the Ixodidae family) and cause human babesiosis, an infection that ranges from asymptomatic to fatal [47]. While *Babesia* spp. tested positive in 13 samples, identification at the species level was not fully parsed out in our qPCR screening. We did specifically test for *Babesia microti*, the primary cause of human babesiosis and a known inhabitant of Pennsylvania, but all samples tested negative [48,49].

*Anaplasma phagocytophilum* is a tick-borne pathogen vectored by *I. scapularis* (endemic to eastern and central United States of America) and has two known strains [50]. The human active variant (*A. phagocytophilum*-ha) was found in two samples and is the only strain of *A. phagocytophilum* in the northeastern United States known to cause human granulocytic anaplasmosis (HGA), a rickettsial infection of neutrophils presenting with serious pathological changes [51]. The second strain, *Anaplasmosis* variant one (otherwise known as *A. phagocytophilum*-v1 or the deer variant), has not been implicated in causing human disease and tested negative in this study's samples [52].

### 4.2 PCR vs. metagenomics identification of tick-borne pathogens

The results of metagenomic analysis generally corresponded to our PCR pathogen screening but provided important additional insights. PCR-positive samples tended to have much higher pathogen abundance, and there was little overlap in pathogen counts between PCR-positive and PCR-negative samples. This agrees with previous studies showing that PCR is highly effective at detecting moderate to high concentrations of known pathogens but may miss those present at low abundance or in mixed infections [53,54].

However, metagenomic analysis detected additional pathogen signatures that PCR did not, consistent with reports that metagenomics has higher sensitivity and broader detection capabilities, particularly for identifying multiple pathogens or novel species in complex microbial communities [30,55]. Furthermore, some PCR-positive samples had only slightly higher pathogen abundance than some PCR-negative samples, underscoring the challenges of threshold-based PCR detection in cases of low-copy DNA or degraded samples. Beyond confirming the presence of pathogens targeted in PCR assays, metagenomic analysis also revealed additional pathogens not included in our PCR screening, suggesting greater sensitivity in picking up low-abundance or degraded nucleic acid fragments that may fall below PCR detection thresholds. This aligns with findings from other tick microbiome studies that emphasized the power of untargeted sequencing approaches to uncover hidden diversity [56,57]. Thus, while PCR screening provides a rapid and cost-effective method for initial pathogen surveillance, it captures only a fraction of the broader microbial landscape that can be uncovered with metagenomics.

Since PCR testing remains more accessible and affordable than metagenomic sequencing, it may not currently be feasible to use metagenomics exclusively for large-scale tick pathogen surveillance. However, for in-depth ecological studies, microbial community profiling, or identification of emerging pathogens, metagenomics offers a much more comprehensive and informative approach when available.

### 4.3 Presence, abundance, and diversity of microbial species and genes

There were no global significant differences in microbial species identified; however, this study elucidated specific microbial assemblages that highlight differences within (alpha) and between (beta) sample diversity. There was significant microbial diversity within the samples when correlating the number of ticks to the alpha metrics Pielou's Evenness and Shannon's Index, where the microbial evenness and richness within each tick sample increased as the number of ticks detected increased.

There was also significant correlation between the percent humidity at the time of sample collection to samples' microbial diversity based on phylogenetic relationships (FIG. 5). Median range percent humidities appeared to cluster more tightly to each other compared to the low and maximum percent humidities that were not clustered, indicating ticks collected in mid-humidities contained microbial consortia that was phylogenetically more similar than the more distantly phylogenetically related low and maximum percent humidities observed. Ticks prefer specific percentages of humidity for their livelihood [58], and this current finding further supports that lineages of microbial consortia on the tick may also thrive or suffer under certain humidity conditions [58].

Additionally, this study observed differences in identified genes within samples via the date, month, location, and time of day samples were collected (Table 4). Samples collected between June and July indicated ticks collected in June had a richer and more even distribution of genes than ticks collected in July (Table 4, SMFIG 2). Ticks endemic to central Pennsylvania are most active between the months of both June and July due to warmer temperatures and humidities, though June is the peak month for Lyme disease [20]. *Ixodes scapularis* ticks are known vectors of Lyme disease and rely on various hosts depending on what stage they are at in their life cycle, though both nymphs and adults have been known to incidentally transmit Lyme disease in humans [59]. *I. scapularis* ticks reach their nymph stage around June, and their much smaller size (compared to their adult counterparts) makes it harder to visibly see them, allowing them to more freely infect unsuspecting human hosts with pathogens [59]. Overall, 711 of the 719 (about 98.9 %) total ticks collected in this study, which included the 96 tick samples selected for further lab processing and analysis, were identified as *I. scapularis*, so it is possible the genes were more rich and even in the June cohort due to the high number of *I. scapularis* adults and nymphs performing certain feeding and host seeking behaviors.

Ticks collected from the southernmost site (71SST) showed lower richness and more unevenly identified genes compared to the northernmost site (PPCT), which was both enriched and evenly distributed. Significant differences between these locations suggest a potential regional effect on microbial function. While local topographic factors such as canopy cover, soil moisture, or disturbance may contribute, the clear contrast between geographically distant sites points toward broader ecological gradients, such as temperature, vegetation, or host community structure [60,61]. Further research combining environmental metadata with microbial profiles is needed to disentangle these regional and local influences.

Time-of-day also influenced microbial function: ticks collected in the late afternoon had less richness and more unevenly identified genes compared to those from the late morning or early afternoon. This may reflect diel changes in tick activity and physiology, as *Ixodes scapularis* is typically more active in lower light but can adapt to daylight when host cues are present [62,63]. Sunlight stability during mid-day hours may promote consistent genes utilized, while variable conditions in the late afternoon could lead to shifts in microbial stress responses or host interaction pathways [56,64]. These patterns suggest that both environmental timing and geography influence microbial functional potential within ticks.

### 4.4 MaAsLin2 biomarker species of pathogen-carrying ticks

Significant multivariate associations were observed between expressed microbial species and several discrete metadata cohorts (Table 5). *Leptomonas seymouri* was enriched in the Leaf Litter cohort of the variable Land Cover when compared to Tall Grass, indicating an ecological preference for leaves over tall grass. *L. seymouri* are monoxenous insect trypanosomatids, or parasitic protozoa that complete their entire life cycle in a single host [65]. This finding is especially interesting because L. *seymouri* are usually found in the orders Siphonaptera and Hemiptera, but they were found in Arachnids (order that includes ticks) in this current study [66], elucidating research gaps regarding L. *seymouri*'s relationship with ticks.

Several tick samples that were PCR positive for a common tick pathogen were also positive for that same pathogen in the metagenomic data, confirming the concordance of PCR and metagenomic detection. For example, samples that tested PCR positive for *Borrelia* (any species) and *Borreliella burgdorferi* were enriched with the bacterial pathogen *Borreliella burgdorferi*. *Borreliella burgdorferi* is transmitted primarily by *Ixodes scapularis*, a hard tick otherwise known as the deer or black-legged tick [67], of which is endemic to the location of tick sample collection [68]. *Borreliella burgdorferi* is of clinical importance as it is the causative agent of Lyme disease in humans, which is the fastest expanding vector-borne disease in the Northern Hemisphere [67]*.*

Another example of the concordance of PCR being confirmed with metagenomic detection was that PCR positive ticks for any species of *Babesia* was also enriched with the taxon *Babesia divergens* in the metagenomic data. The association with *B. divergens* specifically provides additional information beyond that given by the PCR screening. *B. divergens* are intraerythrocytic protozoan parasites, or parasites that transmit a sporozoite (an infective form of the parasite) that enters the host's erythrocytes (red blood cells) [69]. The primary vector of *B. divergens* is *Ixodes ricinus,* a hard tick endemic to Europe [69,70]. Babesiosis features similar clinical presentation to Lyme disease and/or anaplasmosis, which is further complicated in the case of co-infection [71]. While *B. divergens* is transmitted via *I. ricinus* and causes Babesiosis primarily in Europe, the closely related *Babesia microti* causes Babesiosis in the USA, utilizing the endemic Lyme Disease vector *Ixodes scapularis* for transmission [69]. Human Babesiosis occurrence in the USA is rare, and when infection occurs, it primarily affects the elderly, the immunocompromised, and those with asplenia [69,71].

### 4.5 Co-occurring microbial interactions in the tick microbiome

The dominance of positive interactions in the network (FIG. 6) aligns with ecological theories suggesting that microbial communities are shaped by cooperation, syntrophy, or shared environmental constraints [72]. Central taxa like *Lactobacillus* and *Bacteroides*, often linked to host health and microbiome stability, appeared as hubs [73]. The lack of negative correlations may reflect stable environmental conditions or conservative statistical thresholds. While these patterns suggest niche sharing and functional redundancy, co-occurrence does not imply causation. The central and highly connected position of *Rickettsia* supports its likely role as a core endosymbiont rather than a transient or opportunistic pathogen. Prior studies have shown that *Rickettsia* species such as *R. buchneri* and *R. amblyommatis* are vertically transmitted and often reach high prevalence in ticks [61,74]. The observed positive associations may indicate niche complementarity or co-existence rather than competition. However, in other systems, *Rickettsia* has been reported to negatively correlate with other dominant symbionts such as *Coxiella*- or *Francisella*-like endosymbionts, suggesting strain- or host-specific dynamics [75,76]. The absence of *Borrelia*, *Anaplasma*, *Ehrlichia*, and *Babesia* from the network may be due to their typically episodic, horizontally acquired nature [77].

## 5 Conclusions

The findings of this study have pertinent implications to public health education and preventative measures. Clinicians and public health departments may utilize our data to create educational materials for the public (e.g.. brochures, park signs, papers for rangers to hand out) stating which ticks are in the area and the diseases they may carry. Increased awareness of the risks may encourage the public to take appropriate clothing measures while outside and to check themselves for ticks after being outdoors. Clinicians could also offer more specialized and efficient treatment of the tick-borne illnesses if they are more informed about what ticks are in the area and what pathogens and diseases they may be carrying.

### 5.1 Limitations and future directions

This study has several limitations that should inform future research. Although the initial sample size of 96 ticks was somewhat large, only 33 were selected for metagenomic analysis, limiting statistical power. While the sample size was small, the data still provides insight into where these common ticks may be found and what pathogens they may carry in the local central Pennsylvania region. Additionally, the 33 samples selected for metagenomic analysis were only those that tested PCR positive for pathogens, which would have significantly biased the metagenomic microbiome results. For instance, there were a few “statistically significant” results regarding alpha and beta diversity, and MaAsLin2 analysis that implied an environmental condition had an impact on the tick microbiome diversity, or vice versa. Due to the small sample size, it is important to note that correlation does not always imply causation, especially in the confines of this study alone. The authors included this information for future studies to compare against. All samples were collected in Huntingdon County, Pennsylvania, restricting ecological inference to the northeastern U.S. This regional focus aligns with known distributions of *Borreliella burgdorferi* and *Babesia* spp. (51, 52). However, unlike most studies in the region which report *Babesia microti*, our data identified *Babesia divergens* as the dominant *Babesia* species.

To improve statistical resolution and ecological relevance, future studies should increase sample size and geographic scope, ideally incorporating host species (e.g., deer, rodents, pets) and environmental metadata (e.g., drought events, land use). This data can help explain how abiotic or biotic stressors shape tick microbiomes. Longitudinal sampling across seasons and years would also support more robust modeling of temporal changes in pathogen prevalence and microbial composition. The current study only carried out metagenomic analysis on ticks which tested positive for at least one pathogen in the qPCR screening. Future studies should compare the commensal microbiome of pathogen and non-pathogen carrying ticks to see if they differ appreciably. Our study also lacks metatranscriptomic analysis, which would help detect active pathogens and give insight into pathogenic gene expression. Lastly, integrating these data into a computational mapping platform with GIS capabilities could support real-time tracking of tick and pathogen distributions, improving local public health interventions.

## CRediT authorship contribution statement

**Andrew Buonaccorsi:** Writing – review & editing, Writing – original draft, Visualization, Validation, Supervision, Software. **Brittney N. McMullen:** Writing – review & editing, Writing – original draft, Visualization, Validation, Supervision, Project administration, Methodology, Investigation, Formal analysis. **Brie Builder:** Writing – review & editing, Visualization, Validation, Supervision, Methodology, Investigation. **Kelliann Drummond:** Writing – review & editing, Visualization, Methodology, Investigation. **Sarah Halteman:** Writing – review & editing, Writing – original draft, Visualization, Supervision, Methodology, Data curation. **Jeremy Chen See:** Writing – review & editing, Writing – original draft, Visualization, Validation, Supervision, Methodology, Formal analysis, Data curation. **Evan Thomas:** Visualization, Validation, Project administration, Methodology. **Alexa Viands:** Visualization, Validation, Methodology, Investigation. **Sarah Worley:** Supervision, Project administration, Investigation, Funding acquisition, Conceptualization. **Justin R. Wright:** Writing – review & editing, Writing – original draft, Visualization, Validation, Supervision, Methodology, Investigation, Formal analysis, Data curation. **Jill Keeney:** Writing – review & editing, Validation, Supervision, Resources, Project administration, Methodology, Investigation, Funding acquisition, Formal analysis, Data curation, Conceptualization. **Regina Lamendella:** Writing – review & editing, Writing – original draft, Visualization, Validation, Supervision, Resources, Project administration, Methodology, Investigation, Funding acquisition, Formal analysis, Data curation, Conceptualization.

## Declaration of competing interest

The authors declare that they have no competing financial or non-financial interests that could have influenced the work reported in this manuscript. The authors declare no conflicts of interest related to this study. All analyses and interpretations were conducted independently and without influence from any external party. The authors report no proprietary or commercial interest in any product, service, or company mentioned in this article.

## Data Availability

The data used in this study has been made available in the National Center for Biotechnology Information (NCBI) Sequence Read Archive (SRA) database at https://www.ncbi.nlm.nih.gov/sra/PRJNA1283253, under the accession number PRJNA1283253.

## Appendix A Supplementary data

Supplementary material 1 Image 1

Supplementary material 2 Image 2

Supplementary material 3 Image 3
