# Supplementary material for: Metagenomic surveillance of tick-borne pathogens and microbiomes in Huntingdon County, Pennsylvania
Source: One Health. 2025 Dec 18;22:101305. doi: 10.1016/j.onehlt.2025.101305 (PMC12811600; doi:10.1016/j.onehlt.2025.101305)
Supplement: Supplementary file 1 — Supplementary material 1 [file mmc1.docx]

**Supplemental Figure Captions**

SMFIG1: Bar plots showing the relative abundance of tick-borne pathogens versus their PCR results.

The relative abundances of *Babesia divergens* (A), *Borreliella burdorferi* (B), *Borrelia miyamotoi* (C), and *Anaplasma phagocytophilum* (D) in PCR positive (red) and negative (blue) results.

SMFIG2: Boxplots of alpha diversity (pielou’s evenness) for identified genes when considering month (A), date (B), location (C), and time of day (D).

Each boxplot represents the distribution of pielou’s evenness per specified metadata category. The analysis was performed using QIIME 2 (version 2022.11) and visualized via view.qiime2.org.
