# Supplementary figures and images for: Metagenomic surveillance of tick-borne pathogens and microbiomes in Huntingdon County, Pennsylvania
Source: One Health. 2025 Dec 18;22:101305. doi: 10.1016/j.onehlt.2025.101305 (PMC12811600; doi:10.1016/j.onehlt.2025.101305)

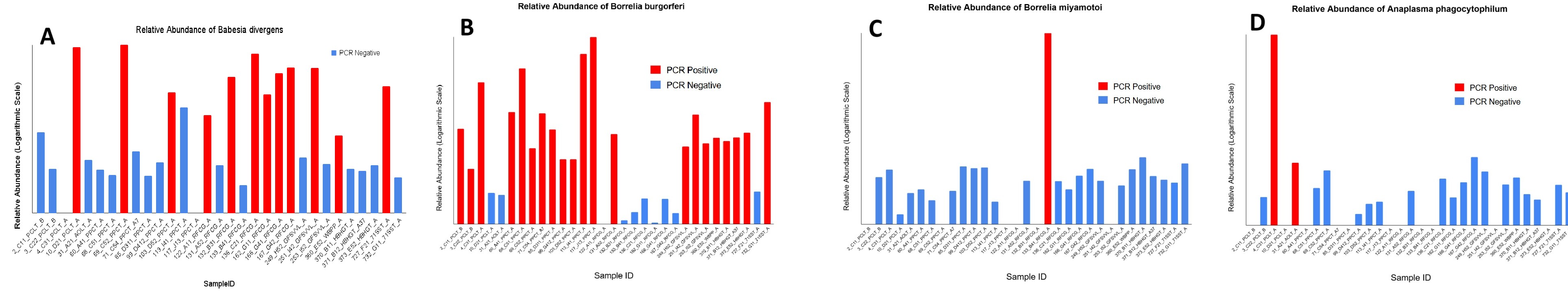

Supplement: Supplementary file 2 — Supplementary material 2 [file mmc2.zip › SMFIG_1.jpeg]

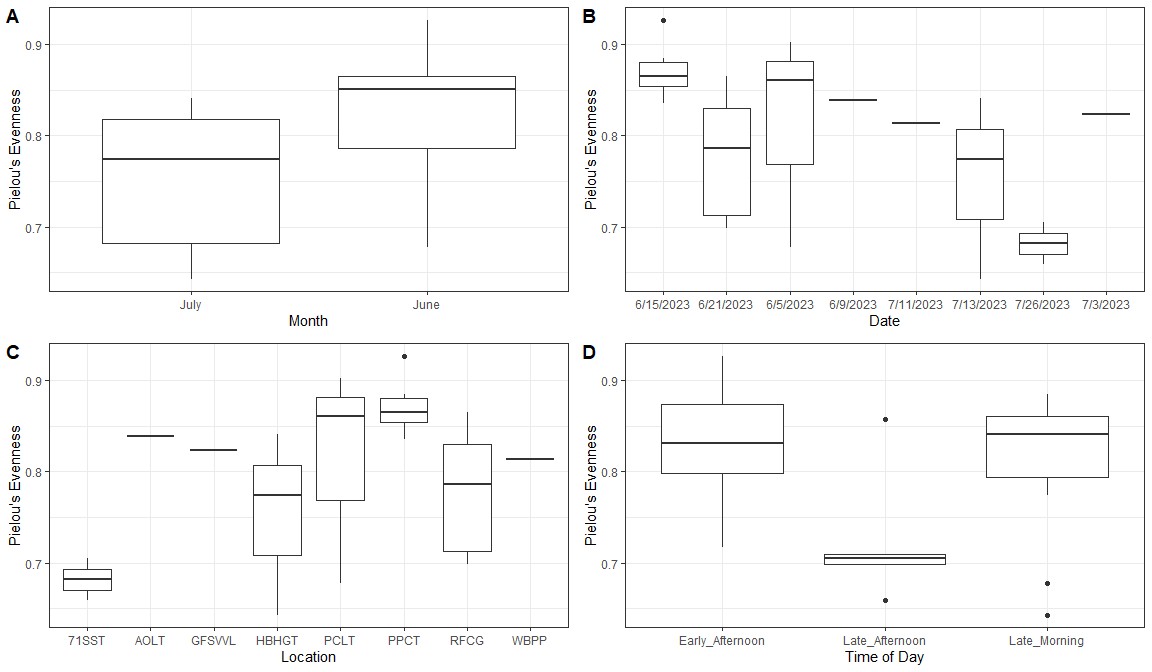

Supplement: Supplementary file 3 — Supplementary material 3 [file mmc3.zip › SMFIG_2.jpeg]
